# Impacts of plastic‐free materials on coral‐associated bacterial communities during reef restoration

**DOI:** 10.1111/1758-2229.13229

**Published:** 2024-01-09

**Authors:** Paige Strudwick, Emma F. Camp, Justin Seymour, Christine Roper, John Edmondson, Lorna Howlett, David J. Suggett

**Affiliations:** ^1^ Faculty of Science, Climate Change Cluster University of Technology Sydney Ultimo New South Wales Australia; ^2^ Wavelength Reef Cruises Port Douglas Queensland Australia; ^3^ King Abdullah University of Science and Technology (KAUST) Thuwal Kingdom of Saudi Arabia

## Abstract

Coral propagation and out‐planting based restoration approaches are increasingly being applied to assist natural recovery of coral reefs. However, many restoration methods rely on plastic zip‐ties to secure coral material which is potentially problematic for the marine environment. Plastic‐free biodegradable alternatives may however pose unique risks to coral‐associated bacterial communities integral to coral health. Therefore, to identify whether biodegradable materials differentially impact coral‐associated bacterial communities we examined *Acropora millepora* coral‐associated bacterial communities during propagation in two experiments on the Great Barrier Reef. Coral fragments were secured to coral nurseries with conventional plastic, metal, or biodegradable (polyester and polycaprolactone) ties. Tie failure and coral‐associated bacterial communities were then characterized over six months. Minimal coral mortality was observed (3.6%–8%) and all ties had low failure rates (0%–4.2%) except for biodegradable polyester ties (29.2% failure). No differences were observed between coral‐associated bacterial communities of fragments secured with different ties, and no proliferation of putatively pathogenic bacteria was recorded. Overall, our findings suggest that reducing reliance on conventional plastic is feasible through transitions to biodegradable materials, without any notable impacts on coral‐associated bacterial communities. However, we caution the need to examine more coral taxa of different morphologies and new plastic‐free materials prior to application.

## Graphical abstract

Plastic‐free alternative tie materials do not cause differential impacts to coral‐associated bacterial communities during coral propagation. Biodegradable polycaprolactone ties performed as well as conventional plastic ties. Consequently, plastic‐free materials are suitable for use in reef restoration and to improve sustainability of practices should be transitioned to in the future.

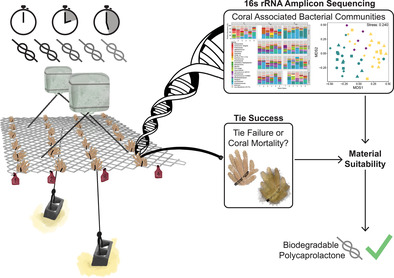

## INTRODUCTION

Coral propagation and out‐planting based restoration practices are accelerating globally to aid conventional reef management approaches (Boström‐Einarsson et al., 2020; Kleypas et al., 2021; Suggett & Van Oppen, 2022). Such practices commonly use an in situ ‘nursery’ phase to increase coral biomass prior to out‐planting material onto bare reef substrate to increase coral cover at rates faster than from natural recovery alone (e.g., Boström‐Einarsson et al., 2020; Howlett et al., 2022; Rinkevich, 2019; Ware et al., 2020). Plastic zip‐ties have particularly become the ‘staple’ for restoration practitioners—primarily for attaching corals to nurseries, but also anchoring corals back to the reef during out‐planting (Boström‐Einarsson et al., 2020; Goergen & Gilliam, 2018)—given their low cost, widespread availability, ease and speed of deployment, and overall lowest chance of fragment dislodgement (Bruckner et al., 2008; Goergen & Gilliam, 2018). However, these benefits of plastic zip‐ties are fast becoming outweighed by the high cost of generation of micro‐ and macro‐plastics (Caron et al., 2018; Huang et al., 2021; Reichert et al., 2022), which pose risks to coral reefs and endemic marine organisms (Bidegain et al., 2018; Lamb et al., 2018; Manfra et al., 2021). Specifically, microbial communities colonizing marine plastic debris (within the ‘plastisphere’) could impact coral microbiomes through direct contact or ingestion and subsequent transfer of foreign microbial communities including pathogens (Hchaichi et al., 2020; Lartaud et al., 2020; Rotjan et al., 2019).

Recently we have shown that coral‐associated microbial communities essential to coral holobiont health can change during propagation and out‐planting practices (Strudwick et al., 2022, 2023), likely from environmental conditions that differ for propagation/out‐planting areas compared to the native reef. Variations in environmental conditions are known to influence coral‐associated bacterial communities (Camp et al., 2020; Kelly et al., 2014; Maher et al., 2019; McDevitt‐Irwin et al., 2017; Ziegler et al., 2017, 2019) and coral propagation structures could conceptually facilitate the proliferation of disease (Moriarty et al., 2020). However, how potential environmental changes induced by the materials used during the propagation process (e.g., metal structures and/or fixing devices, such as plastic zip‐ties) impact propagated corals remains untested. Consequently, while transitioning to plastic‐alternatives in reef construction and engineering (Manfra et al., 2021; Nauta et al., 2022)—including intervention practices—is a matter of urgency (Boström‐Einarsson et al., 2020; Ceccarelli et al., 2020), plastic alternatives such as metal or biodegradable plastics may present microbial risks to coral reefs. Biodegradable materials can have enhanced biofouling (Dussud et al., 2018; Peng et al., 2022) through high microbial affinity (Peng et al., 2022) and microbial driven break‐down (Gan & Zhang, 2019; Manfra et al., 2021) and could result in proliferation of putative pathogens within the coral microbiome (Ceccarelli et al., 2020; Dussud et al., 2018; Hchaichi et al., 2020; Zettler et al., 2013). Indeed, increased transfer of putatively pathogenic *Vibrio* spp. and trace metals—linked to increased pathogenicity (Rubio‐Portillo et al., 2020)—from plastic‐free biodegradable materials to marine organisms can occur (catfish, Jang et al., 2022). As such, use of plastic alternatives could arguably negatively impact coral bacterial communities despite best intentions for a more environmentally positive attachment solution.

While stainless‐steel metal ties have long been available, biodegradable zip‐ties have only more recently become commercially available (Haider et al., 2019). Biodegradable zip‐ties are fabricated from polymers that degrade under prolonged exposure to UV light, heat, moisture and microbial metabolic activity through several stages including biodegradation, bio‐fragmentation, assimilation and mineralization (Delacuvellerie et al., 2021; Lucas et al., 2008). Surface marine bacterial communities of both degradable and non‐degradable plastics show similarities after short time frames (~80 days) (Delacuvellerie et al., 2021); however, how these ‘plastisphere’ microbial communities evolve over longer periods remains unexplored (Delacuvellerie et al., 2023). It is known that biodegradable materials have high microbial affinity and biomass (Peng et al., 2022) and can transfer putative pathogenic bacteria (e.g., *Vibrio* spp.) to marine organisms (Jang et al., 2022). As such, it is plausible to expect evolution of microbial communities in the biodegradable plastisphere to differ from that of conventional plastic (Delacuvellerie et al., 2021; Dussud et al., 2018) and potentially include proliferation of putatively pathogenic bacteria of the *Vibrio* genus. Therefore, biodegradable material zip‐ties may impact coral‐associated bacterial communities differentially to conventional plastic, especially as coral propagation in nursery environments already promotes shifts in coral‐associated bacterial communities for some coral species (Strudwick et al., 2022).

Here we compared performance of zip‐ties fabricated from plastic and plastic‐free materials for coral propagation processes under the hypotheses that: (i) differences in zip‐tie material will shape coral‐associated bacterial communities and (ii) coral fragments attached with biodegradable zip‐ties will have increased relative abundance of bacteria that are putative coral pathogens from the *Vibrio* genus. To test these hypotheses, and to inform coral reef restoration practitioners of the suitability of plastic‐alternatives, we tracked the microbiome of coral fragments attached to in situ nurseries with five different zip‐tie materials, via two consecutive experiments each lasting 6‐months.

## EXPERIMENTAL PROCEDURES

### Sampling location and experimental design

Experiments were conducted at coral nursery sites ‘Blue Lagoon’ and ‘Mojo’ located at Opal Reef (16°12′18″ S 145°53′54″ E), which is a 24.7 km^2^ reef situated on the northern Great Barrier Reef (GBR) (detailed in Howlett et al., 2021; Suggett et al., 2019). All nursery sites at Opal Reef consist of multiple floating frames located at depths of 5–6 m on sand immediately adjacent to the reef (detailed in Howlett et al., 2021) (Figure S1). For each experiment two dedicated nursery frames were installed and conditioned in situ for a period of at least 2 weeks prior to beginning the experiment. All operations and sampling were conducted under permits G21/45224.1 and G20/43740.1.

Coral fragments were harvested from donor colonies of *Acropora millepora* and secured to nursery frames via three different attachment materials (Figure S2). The first experiment was conducted from August 2020–February 2021 at site ‘Blue Lagoon’, Opal Reef, which is subject to tidal currents due to close proximity to a deep‐water channel (see Howlett et al., 2021; Suggett et al., 2019), where corals have previously been demonstrated to achieve good recovery from bleaching and storm damage (Edmondson pers. obs). For this experiment, the three ties compared were plastic (black Nylon‐66), biodegradable A (Poly‐1,4‐butanediol Succinate, supplier gocableties.co.uk) and metal (316 grade stainless steel) (full specifications; Table S1). After fastening corals, excess tie was cut off, apart from for the metal tie where this was not possible.

A second experiment was conducted from February–August 2022 at site ‘Mojo’, Opal Reef, which is 150 m from Blue Lagoon and shares topographical characteristics and similar natural recovery to past disturbance events. A different combination of ties were used for this experiment, including plastic (‘natural’ colour Nylon‐66), biodegradable material B (Polycaprolactone, supplier rapstrap.com) and Rapstrap soft plastic (Polyurethane Elastomer, supplier rapstrap.com) (full specifications; Table S1). Excess tie was cut after fastening corals (Figure S2a,b). Although the timing for the two experiments differed, the same Nylon‐66 plastic ties were used and hence provided a control group.

**FIGURE 1:**
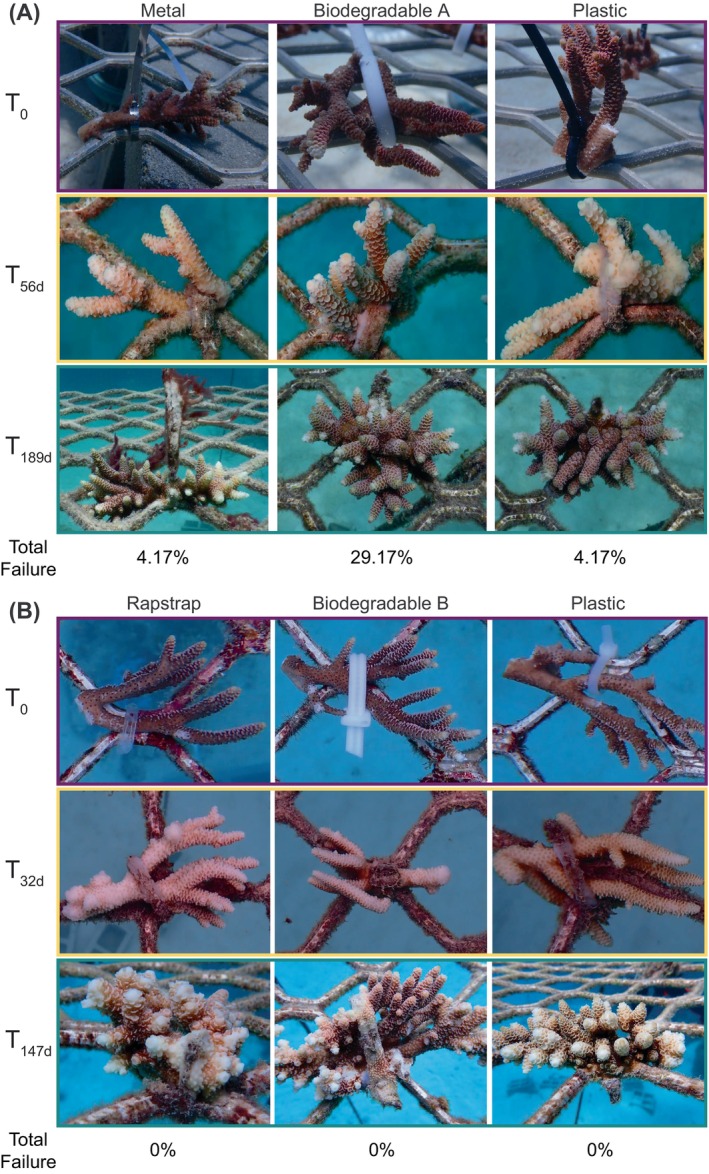
Coral fragments of *Acropora millepora* secured in nurseries at with three different attachment materials at (A) Blue Lagoon over 6 months from August 2020 to February 2021 and (B) at Mojo over 6 months from February 2022 to August 2022 at Opal Reef, northern Great Barrier Reef. Note different fragments are shown at each time point for any given tie type.

The sampling design for both experiments consisted of three (start, intermediate and end) sampling events; however, the respective timing of intermediate and end sampling events differed due to logistical and reef access constraints. At Blue Lagoon, six donor colonies (≥55 cm diameter) were identified on the reef adjacent to nurseries and marked with cattle tags, with each colony representing a biological replicate. In total 13 fragments (≤5 cm) were harvested from each donor colony using wire cutters and transported in a sterile zip‐lock bag with seawater by a diver to nursery frames located 10–20 m away. Harvested fragments from three of six donor colonies were taken to frame one and the remainder were taken to frame two (3 m apart) to account for potential frame effects. Fragments from an individual donor colony were divided into three groups of four and immediately attached to nursery frames using one of the three tie types (Figures 1A and S3). At Mojo, five *A. millepora* donor colonies were identified and marked with cattle tags, in total 19 (≤5 cm) fragments were harvested from each donor colony divided into three groups of six and immediately attached to nursery frames using one of the three tie types (again spread over two nursery frames to account for frame effect). Previous studies have measured increased abundance of putatively pathogenic microbes in less than 45 days on marine plastic debris (Zettler et al., 2013) and/or changes in coral‐associated bacterial communities for coral propagated in nurseries for 4 months (Strudwick et al., 2022) as such an intermediate sampling point and five to six month sampling point were used to capture initial and/or later onset changes in coral‐associated bacterial communities.

At the time of harvesting fragments from donor colonies, one fragment from each donor colony was placed in an individual sterile zip‐lock bag and taken to the operations vessel (*Wavelength 4*) and preserved in RNA*later* for donor colony ‘time zero’ (T_0_) bacterial community characterization (Figure S3a,b). In the first experiment (Blue Lagoon, August 2020–February 2021), nursery fragments and donor colonies were re‐sampled at 56 days (T_56d_) and 189 days (T_189d_) (Figure S3a,b). In the second experiment (Mojo, February 2022–August 2022), nursery fragments and donor colonies were re‐sampled at 32 days (T_32d_) and 147 days (T_147d_) (Figure S3a,b).

At each time point, coral fragments were removed from the nursery frame by detaching the cable tie (or fragmented using wire clippers where fragments had self‐attached to the nursery frame) and from the original marked donor colony on the reef using wire clippers and preserved by submersion in RNA*later* in sterile falcon tubes. In the first experiment, 54 samples were collected for bacterial community analysis: (i) nursery fragments: six replicates (three from each nursery frame) x three attachment materials x two time points (*n* = 36) plus (ii) donor colonies: six replicates x three time points (*n* = 18). In the second experiment, 45 samples were collected for bacterial community analysis: (i) nursery fragments: five replicates (2–3 from each nursery frame) × three attachment materials x two time points (*n* = 30) plus (ii) donor colonies: five replicates × three time points (*n* = 15).

### Quantification of failure and coral mortality

At each sampling time point, counts were conducted to record the number of (i) live coral fragments present with tie, (ii) live coral fragment present without tie, (iii) tie present with coral missing, (iv) tie and coral missing and (v) dead coral. Tie failure was considered to have occurred when either live coral fragments were present without tie, when a tie was present without coral fragment, or when both tie and coral fragment were missing. At the end of the study, tie failure rate percentage (%) was calculated by dividing the total number of failed ties by the total number of coral fragments attached at T_0_ and multiplying by 100.

### DNA extraction, 16S rRNA amplicon sequencing and bioinformatics

DNA was extracted from coral tissue isolated using an airbrushing technique (see Supplementary Methods) as per Strudwick et al. (2022) using DNeasy Blood and Tissue kit (Qiagen) following the Manufacturer's protocol (July 2020 version) with a total elution volume of 40 μL. Extracted DNA was stored at −30°C for 1 week prior to 16S rRNA amplicon sequencing. The hypervariable V3 and V4 regions of the bacterial 16S rRNA gene were amplified using the primers 341F (5′‐CCTAYGGGRBG‐CASCAG‐3′) and 805R (5′‐GACTACHVGGGTATC‐TAATCC‐3′) (Klindworth et al., 2013), prior to sequencing on the Illumina MiSeq platform (Ramaciotti Centre for Genomics, Sydney, NSW, Australia). Raw data files in FASTQ format were deposited in NCBI Sequence Read Archive (SRA) under Bioproject number PRJNA945487.

For both experiments raw demultiplexed sequencing data were analysed using the Quantitative Insights into Microbial Ecology (QIIME 2, version 2020.6) platform (Callahan et al., 2016). The DADA2 plugin was used to denoise the data (Callahan et al., 2016) and taxonomy was assigned using the classify‐sklearn classifier (Pedregosa, 2013) against the SILVA v138 database. Amplicon sequence variants (ASVs) corresponding to chloroplast or mitochondria were removed from the data set. To remove contaminants identified in ‘kit blank’ extractions, ASVs were removed with (i) >5% relative abundance in kit blank samples or (ii) greater relative abundance than total coral samples and (iii) that have been previously reported as contaminants of laboratory reagents (Weyrich et al., 2019). Overall, 11 and 13 ASVs were removed from the August 2020–February 2021 and February 2022–August 2022 data, respectively. Prior to diversity analyses, one sample was removed from the August 2020–February 2021 data set due to poor sequencing output providing low read numbers after quality filtering and contaminant removal. For beta diversity analyses, the raw read ASV table was converted to relative abundances, scaled to 20,000 (McKnight et al., 2019) and square root transformed.

### Statistical analysis

To identify whether bacterial community structure differed after corals were placed in the nursery, beta diversity patterns of nursery corals at both intermediate (T_32d/56d_) and end (T_147d/189d_) time points and donor colonies at time of harvesting fragments (T_0_) were analysed using the Bray–Curtis dissimilarity distance metric and patterns were visualized using non‐metric multidimensional scaling (n‐MDS) plots with the *ggplot2* package (Wickham et al., 2016) in R version 4.2.3 (R Core Team, 2013). Further analysis was conducted to identify differences in associated bacterial community structure between nursery fragments attached with different materials (fixed effect = tie type). Homogeneity of multivariate dispersions was tested with the *betadisper* function of the *vegan* package in R version 4.2.3 (Oksanen et al., 2022; R Core Team, 2013). Differences in beta diversity were tested for significance with permutational multivariate analysis of variance (PERMANOVA; perm = 999) and (if significant) subsequent pairwise post‐hoc of Bray–Curtis dissimilarities using the *adonis2* function of the *vegan* package (Oksanen et al., 2022) and *pairwiseAdonis* function, respectively (Martinez Arbizu, 2020). For pairwise comparisons p‐values were subsequently adjusted by applying a Benjamini and Hochberg (a.k.a. False Discovery Rate) correction to account for multiple comparisons, all *p*_*adj*_ values <0.05 were considered significant. To test for possible confounding effects from treatments we ran *adonis* with ‘Site’ (e.g., reef, nursery 1, and nursery 2) as a random factor.

To test whether coral fragments secured with biodegradable materials (A or B) harboured bacterial communities with higher relative abundance of putative coral pathogens from the *Vibrio* genus, *Vibrio* genus ASVs were grouped, average relative abundance for each tie type and time point was calculated and compared with a Kruskal–Wallis test to assess significance.

## RESULTS AND DISCUSSION

### Impact of tie‐material on coral propagule survival

Survival of coral propagules was high across all tie types (including conventional plastic). Specifically, only two fragments (that were attached with plastic) out of 162 total fragments exhibited mortality (Tables S2 and S3). Almost all fragments had begun to overgrow their respective ties after 32–56 days and no signs of disease were recorded (Figure 1A,B). Although failure of some kind (e.g., loss of coral or loss of tie) was observed for most ties, low failure was observed across both experiments for all tie materials (0%–4.17%), except for biodegradable material A that had the highest failure rate (29.17%) (Table S3). The high failure rate observed in biodegradable material A ties potentially resulted from early compromise—presumably via degradation—indicating that using this material for other coral species (with slower growth) would likely have even higher failure. As such our results suggest that biodegradable material B (polycaprolactone) zip‐ties appeared more suited for securing coral in nursery propagation; however, wider species‐specific investigation is required to ensure tie time‐to‐failure exceeds coral time‐to‐attachment prior to widespread use.

### Impact of tie‐material on coral‐associated bacterial communities

**FIGURE 2:**
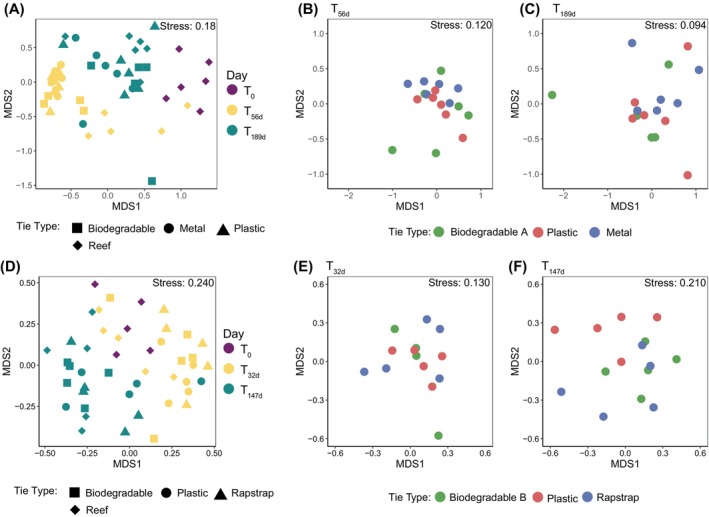
Bacterial community structure of donor colonies and nursery fragments over time. No nursery fragments are included at T_0_ as they had not yet been deployed. Bacterial community structure and relative dispersion of the microbial communities of (A) donor colonies at time of harvesting (T_0_) and nursery fragments and donors at T_56d_ and T_189d_, and just nursery fragments at (B) T_56d_ and (C) T_189d_ for the first experiment. Bacterial community structure and relative dispersion of (D) donor colonies at time of harvesting (T_0_) and nursery fragments and donor colonies at T_32d_ and T_147d_ and just nursery fragments at (E) T_32d_ and (F) T_147d_ in the second experiment. Plots are based on non‐metric multidimensional scaling (n‐MDS) of Bray–Curtis distances of bacterial community structure.

For the first experiment assessing biodegradable material A, the structure of coral‐associated bacterial communities of all coral propagules after 56 days in the nursery—regardless of attachment type—significantly changed from that at time of harvesting fragments (T_0_) (PERMANOVA_Bray‐curtis_, *p*_adj_ < 0.05, Figures 2A, S5 and Supplementary Data S1). For the second experiment assessing biodegradable material B, ‘Rapstrap’ and plastic ties, the structure of coral‐associated bacterial communities of propagules attached with plastic and Rapstrap ties changed after 32 days (T_32d_) compared to time of harvesting fragments (T_0_) (PERMANOVA_Bray‐curtis_, *p*_adj_ < 0.05, Figures 2D, S6 and Supplementary Data S1). In contrast, bacterial community structure of coral fragments attached to nurseries with biodegradable (B) ties differed from time of harvesting fragments (T_0_) after a longer period of time in the nursery (147 days; T_147d_) (PERMANOVA_Bray‐curtis_, *F* = 1.283, *p*_adj_ = 0.022, Figures 2D, S6 and Supplementary Data S1). For both experiments, treatment (e.g., reef, nursery 1, and nursery 2) was not identified as a confounding factor for the PERMANOVA results. Changes in coral‐associated bacterial communities after transfer from native reef to coral nurseries observed in this study are consistent with previous observations for *A. millepora* during nursery propagation on the GBR (Strudwick et al., 2022) and are typical of this coral genus when transported between distinct environments (Haydon et al., 2021; Strudwick et al., 2023; Ziegler et al., 2019).

In line with previous studies highlighting variability of *Acropora* spp. bacterial communities over time and space (Haydon et al., 2021; Strudwick et al., 2023; Ziegler et al., 2019), coral fragments growing in nurseries over the course of both experiments exhibited temporal changes in associated bacterial community structure; specifically, for experiment one from 56 days (T_56d_) to 189d (T_189d_) (PERMANOVA_Bray‐curtis_ *p*_adj_ < 0.05, Figure 2A, S5 and Supplementary Data S1), and for experiment two from 32 days (T32d) to 147 days (T147d) (PERMANOVA_Bray‐curtis_ *p*_adj_ < 0.05, Figures 2D, S6 and Supplementary Data S1). However, in both experiments there were no differences between the structure of bacterial communities of fragments in nurseries attached with different ties at any time; specifically, in experiment one for plastic, biodegradable (A) and metal ties at 56 days (T_56d_) and 189 days (T_189d_) (PERMANOVA_Bray‐curtis_ *p*_*adj*_ > 0.05, Figures 2B,C, S5 and Supplementary Data S1) and in experiment two for plastic, biodegradable (B) or Rapstrap ties at 32 days (T_32d_) and 147 days (T_147d_) (PERMANOVA_Bray‐curtis_ *p*_*adj*_ > 0.05, Figures 2E,F, S6 and Supplementary Data S1). While different materials are suggested to be colonized by distinct microbial communities (Caruso, 2020) there were no differential impacts to coral bacterial communities between tie types. Biodegradable materials A and B did not differentially impact coral bacterial communities suggesting high suitability for use in these nursery‐based reef restoration activities.

### *Abundance of putatively pathogenic* Vibrio *spp.*

Enriched populations of putatively pathogenic *Vibrio* spp. have been observed on plastic and biodegradable plastic materials in marine environments (Dussud et al., 2018; Zettler et al., 2013). However, in our study we observed no differences in the relative abundance (RA) of *Vibrio* spp. between coral fragments secured with biodegradable (A) or plastic ties (mean RA = 0.88%, mean RA = 1.21%, respectively) and donor colonies (mean RA = 0.26%) or other nursery fragments secured with metal ties (RA range = 0.57%) (Kruskal–Wallis, *p* > 0.05, and Figure S4a). Similarly, in the second experiment RA of *Vibrio* spp. did not differ between coral fragments secured with biodegradable (B), plastic or Rapstrap ties (mean RA = 0.28%, mean RA = 0.16%, mean RA = 0.54%, respectively) and donor colonies (mean RA = 0.12%) (Wilcoxon rank sum, *p* < 0.05, and Figure S4b). Consequently, there was no evidence to suggest that using the biodegradable materials tested in this study would increase abundance of putatively pathogenic taxa in coral propagule microbiomes.

### Conclusion

Reducing reliance on plastic materials and optimizing protocols is key to advancing coral propagation and out‐planting approaches. However, little is known about the impact of changing the materials used to fasten corals to artificial propagation structures. Here we examined whether biodegradable materials used to secure coral to nurseries differentially impact the coral‐associated bacterial communities compared to conventional plastics. In contrast to our hypotheses that tie material would influence coral‐associated bacterial communities (and that biodegradable ties would cause higher putative pathogen loads), we found no significant change in coral bacteria communities that could be explained by the tie material. Generally plastic‐free alternatives had low failure rates and high coral survival similar to conventional plastic except for biodegradable material A. However, how well these materials perform for other coral taxa with particularly different growth rates and morphologies needs to be tested. Further, as highlighted here—not all biodegradable materials have equal failure—and as more biodegradable options become available it will be essential to quantify their respective ‘life spans’ in marine environments to ensure degradation does not occur prior to coral self‐attachment. In conclusion, in this study we show that biodegradable materials do not differentially impact associated bacterial communities of fragments grown in coral nurseries and suggest that transitions from conventional plastic to particular biodegradable alternatives while avoiding impacts to coral‐associated bacterial communities is possible.

## AUTHOR CONTRIBUTIONS

**Paige Strudwick:** Conceptualization (equal); data curation (equal); formal analysis (equal); investigation (equal); methodology (equal); project administration (equal); validation (equal); visualization (equal); writing – original draft (equal). **Emma F. Camp:** Conceptualization (equal); funding acquisition (equal); methodology (equal); project administration (equal); supervision (equal); writing – review and editing (equal). **Justin Seymour:** Conceptualization (equal); funding acquisition (equal); methodology (equal); resources (equal); supervision (equal); writing – review and editing (equal). **Christine Roper:** Investigation (equal); methodology (equal); project administration (equal); writing – review and editing (equal). **John Edmondson:** Investigation (equal); methodology (equal); project administration (equal); writing – review and editing (equal). **Lorna Howlett:** Investigation (equal); methodology (equal); project administration (equal); writing – review and editing (equal). **David Suggett J:** Conceptualization (equal); funding acquisition (equal); investigation (equal); methodology (equal); project administration (equal); resources (equal); supervision (equal); writing – review and editing (equal).

## CONFLICT OF INTEREST STATEMENT

The authors declare no conflicts of interest.

## Supporting information


**Data S1.** Supporting information.

**Data S2.** Supporting information.

## Data Availability

The datasets generated during and/or analysed during the current study are available in the NCBI Sequence Read Archive (SRA) under Bioproject number PRJNA945487 (https://www.ncbi.nlm.nih.gov/bioproject/PRJNA945487) or within the Supplementary Data sheets provided.
