# Supplementary material for: Impacts of plastic‐free materials on coral‐associated bacterial communities during reef restoration
Source: Environ Microbiol Rep. 2024 Jan 9;16(1):e13229. doi: 10.1111/1758-2229.13229 (PMC10866064; doi:10.1111/1758-2229.13229)
Supplement: Supplementary file 1 — Data S1. Supporting information. [file EMI4-16-e13229-s001.docx]

**Impacts of plastic‐free materials on coral‐associated bacterial communities during reef restoration**

Paige Strudwick^1^* (ORC ID: 0000-0003-3053-5041), Emma F. Camp^1^ (ORC ID: 0000-0003-1962-1336), Justin Seymour^1^ (ORC ID: 0000-0002-3745-6541), Christine Roper^1^ (ORC ID:

0000-0001-6128-9387), John Edmondson^2^ (ORC ID: 0000-0001-8328-5600), Lorna Howlett^1^ (ORC ID: 0000-0001-8095-1647), David J Suggett^3^ (ORC ID: 0000-0001-5326-2520)

^1^ University of Technology Sydney, Climate Change Cluster, Faculty of Science, Ultimo, NSW, 2007, Australia

^2^ Wavelength Reef Cruises, 6/43 Macrossan St., Port Douglas, QLD, 4877, Australia

^3^ King Abdullah University of Science and Technology (KAUST), Thuwal 23955-6900, Kingdom of Saudi Arabia

*Correspondence: paige.strudwick@uts.edu.au

## Supplementary methods

All samples were held at 4 °C for four to six days during transportation from the study site to the laboratory. Once in the laboratory, RNA*later*was thoroughly removed and samples were preserved at –80 °C for up to six months until DNA was extracted. Prior to DNA extraction, samples were thawed and rinsed with autoclaved phosphate-buffered saline (PBS) (3X, pH 7.4), and coral tissue was removed from the skeleton via air brushing into 4 mL of PBS. The tissue slurry was divided across two 2 mL micro centrifuge tubes and centrifuged at 8000 rpm for 5 min. The supernatant was removed, and the tissue pellet was stored at –80°C for one to two weeks until DNA extraction. DNA was extracted from approximately 200 µl of the coral tissue pellet using a DNeasy Blood and Tissue kit (Qiagen) following the Manufacturer’s protocol (July 2020 version) with a total elution volume of 40 µL. Kit “blank” samples were included in DNA extractions to identify any laboratory contaminants. Extracted DNA was quality checked and the concentration was quantified using a NanoDrop spectrophotometer.

## Supplementary Figures


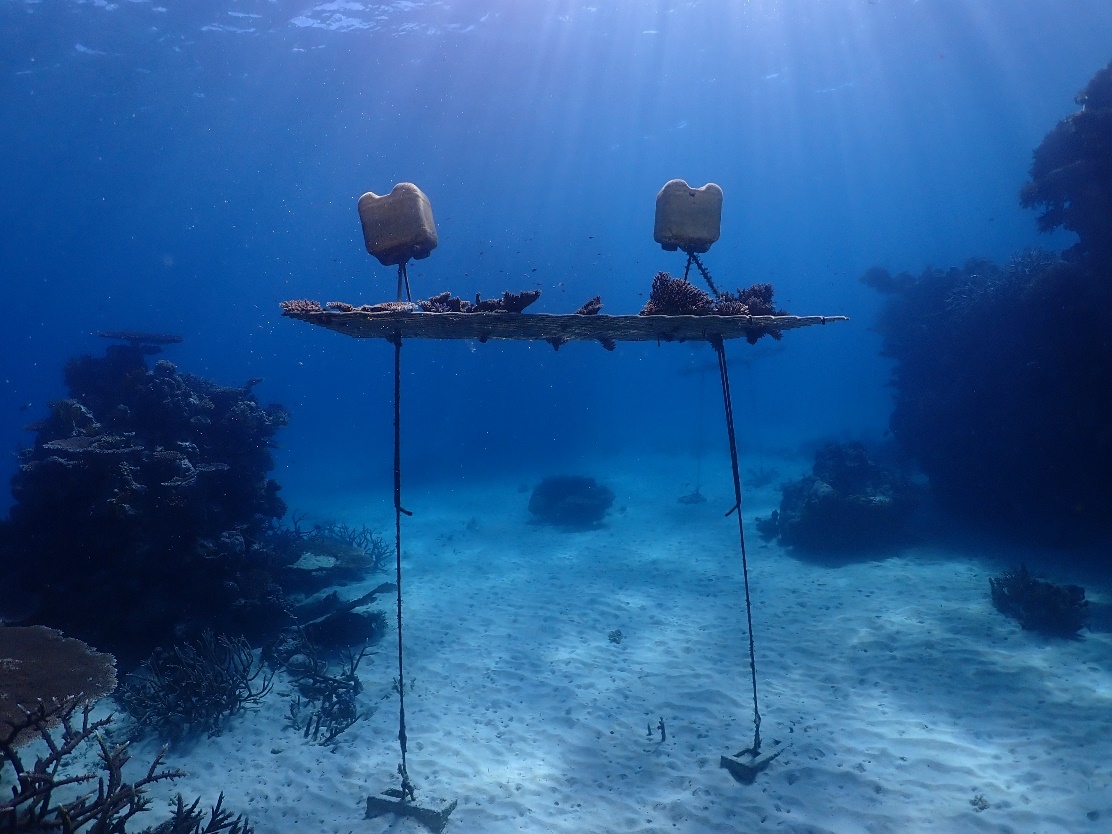


***Fig. S1.*** *Example of coral nursery at Opal Reef, photo: John Edmondson.*


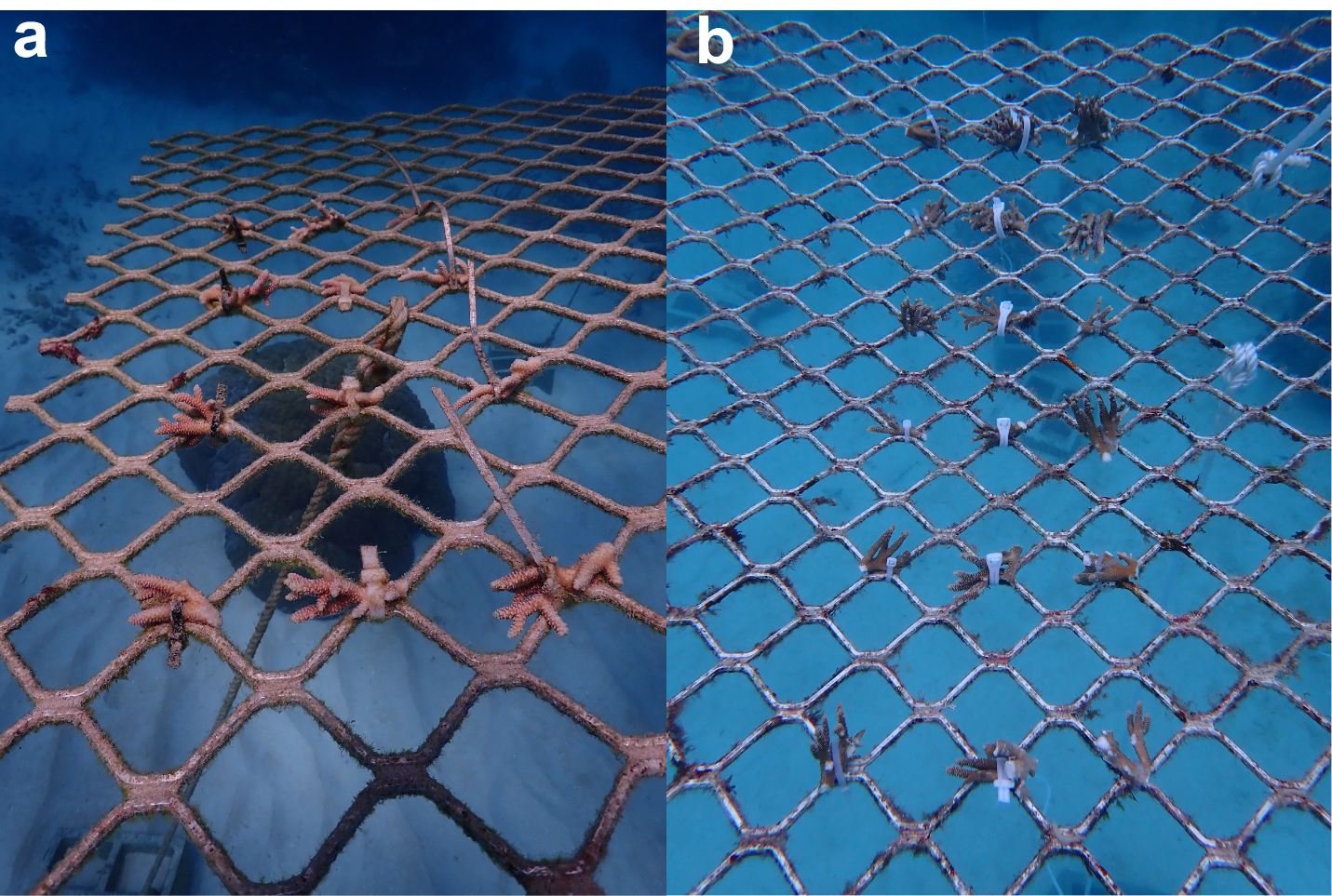
 ***Fig. S2.*** *Fragments of A. millepora in coral nurseries at Opal Reef, northern GBR. Coral fragments are secured with (from left to right) (****a****) plastic, biodegradable material A and metal ties in September 2020; and (****b****) plastic, biodegradable material B and Rapstrap ties in February 2022.*


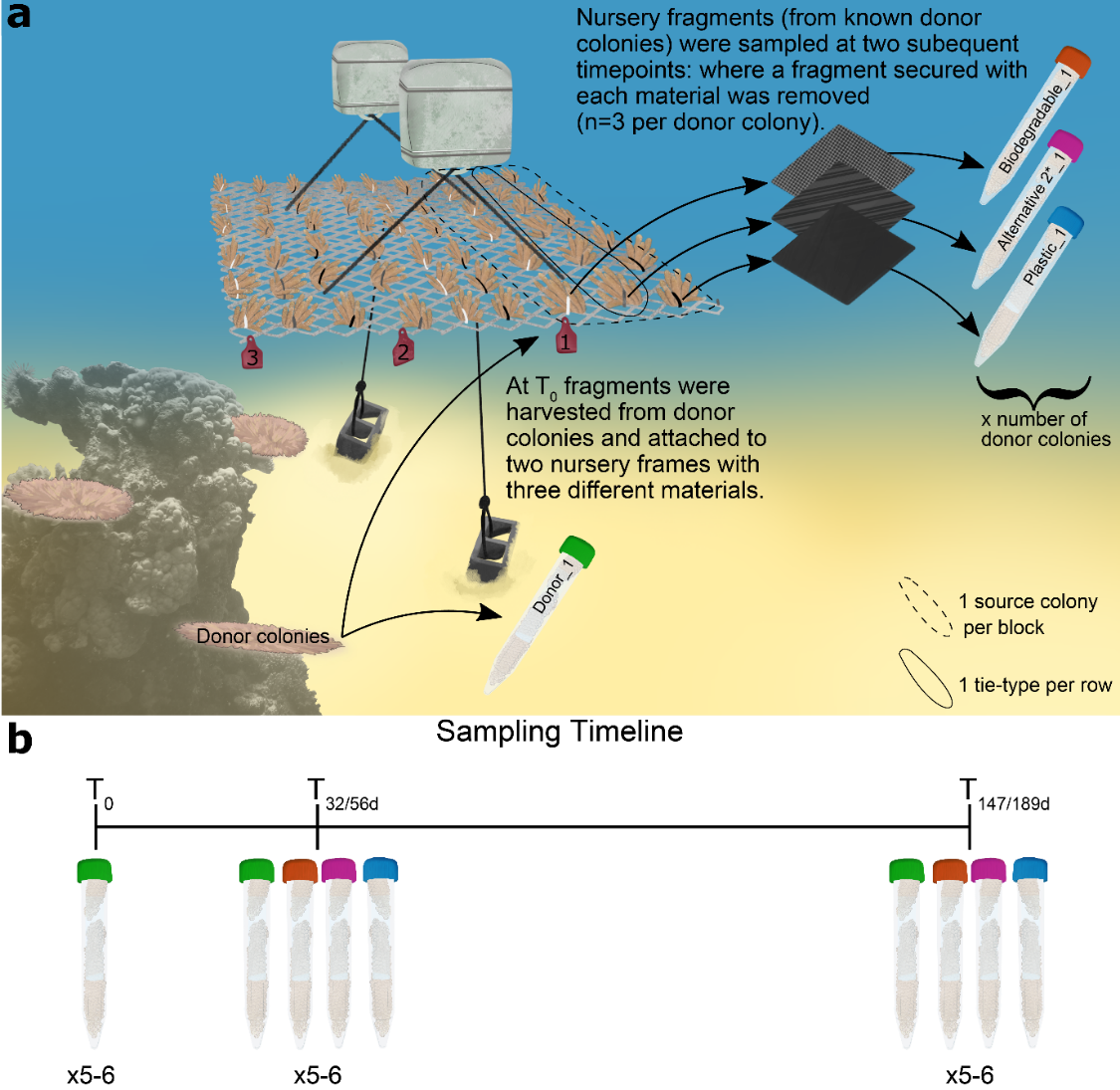


***Fig. S3.*** *Illustration of (****a****)* *the experimental design and nursery set up and (****b****) sampling timeline of the two experiments.*

**
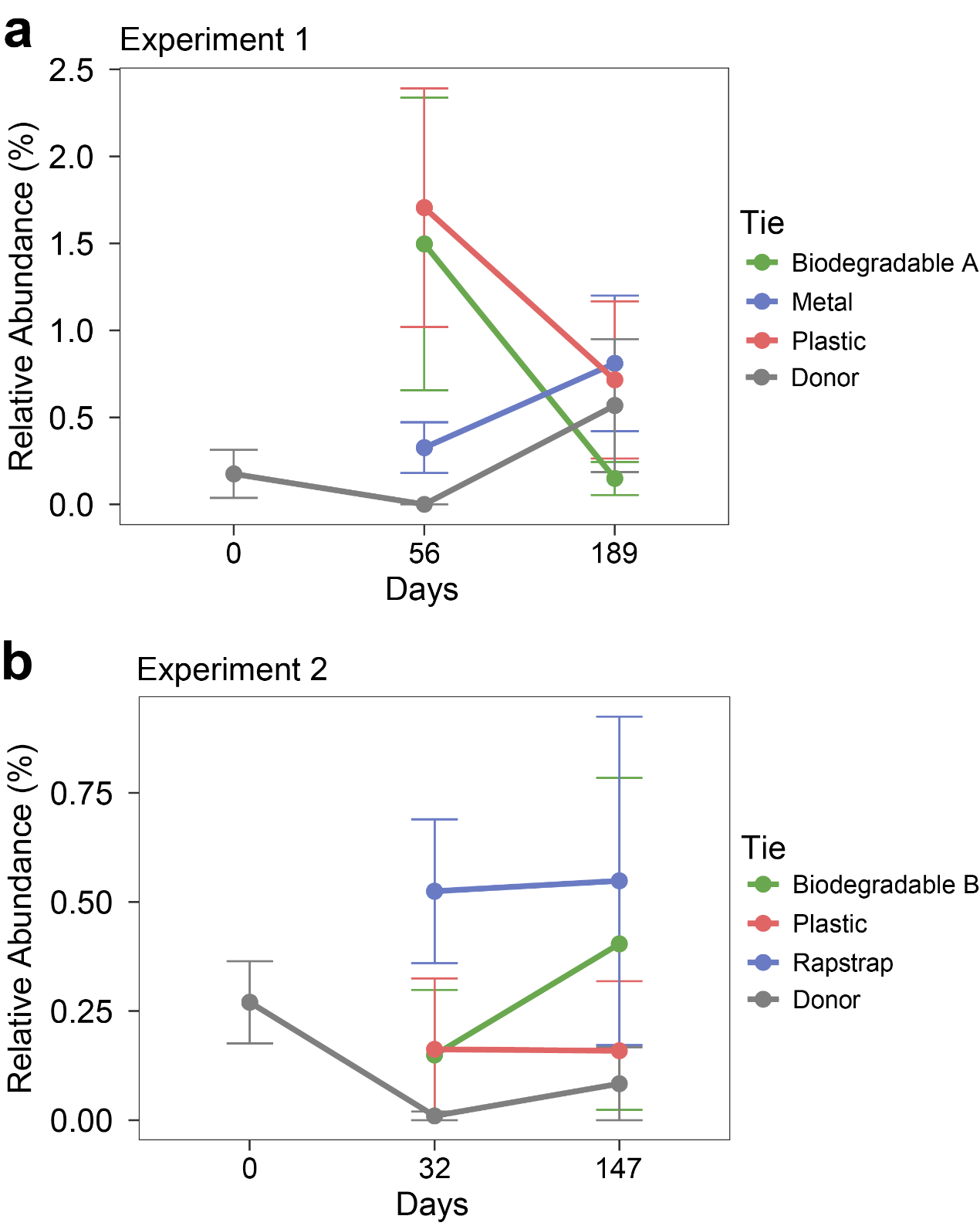
**

**Fig S4**. *Relative abundance of bacteria within the Vibrio genus for Acropora millepora donor colonies and fragments within a coral nursery over time; in two studies assessing (****a****) biodegradable material A, plastic and metal zip-ties and (****b****) biodegradable material B, plastic and Rapstrap zip-ties to secure coral fragments (Source Data provided as Supplementary Data S2.).*


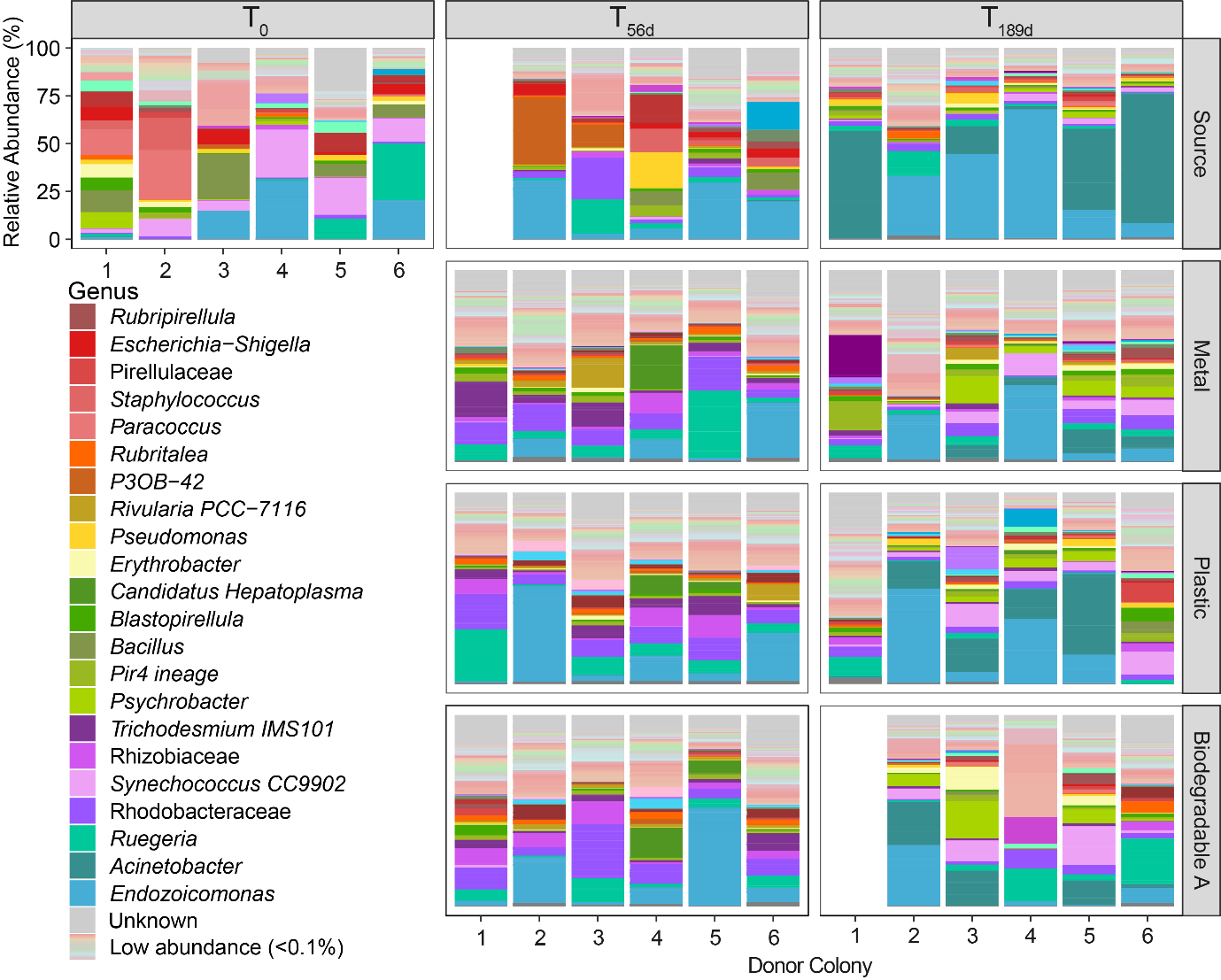


**Fig S5.** *Bacterial community composition (relative abundances) by genus* of donor colonies at the start of the experiment (T_0_) and fragments after 56 and 189 days (from August 2020 to February 2021) within a nursery secured with different materials; metal, plastic and biodegradable material A. Pastel colours represent genera with an average relative abundance of < 0.1 % in all samples, full legend provided as supplemental data (Supplementary Data S3a.) *non-italic text indicates family where – taxonomic resolution to genus level was unavailable.*


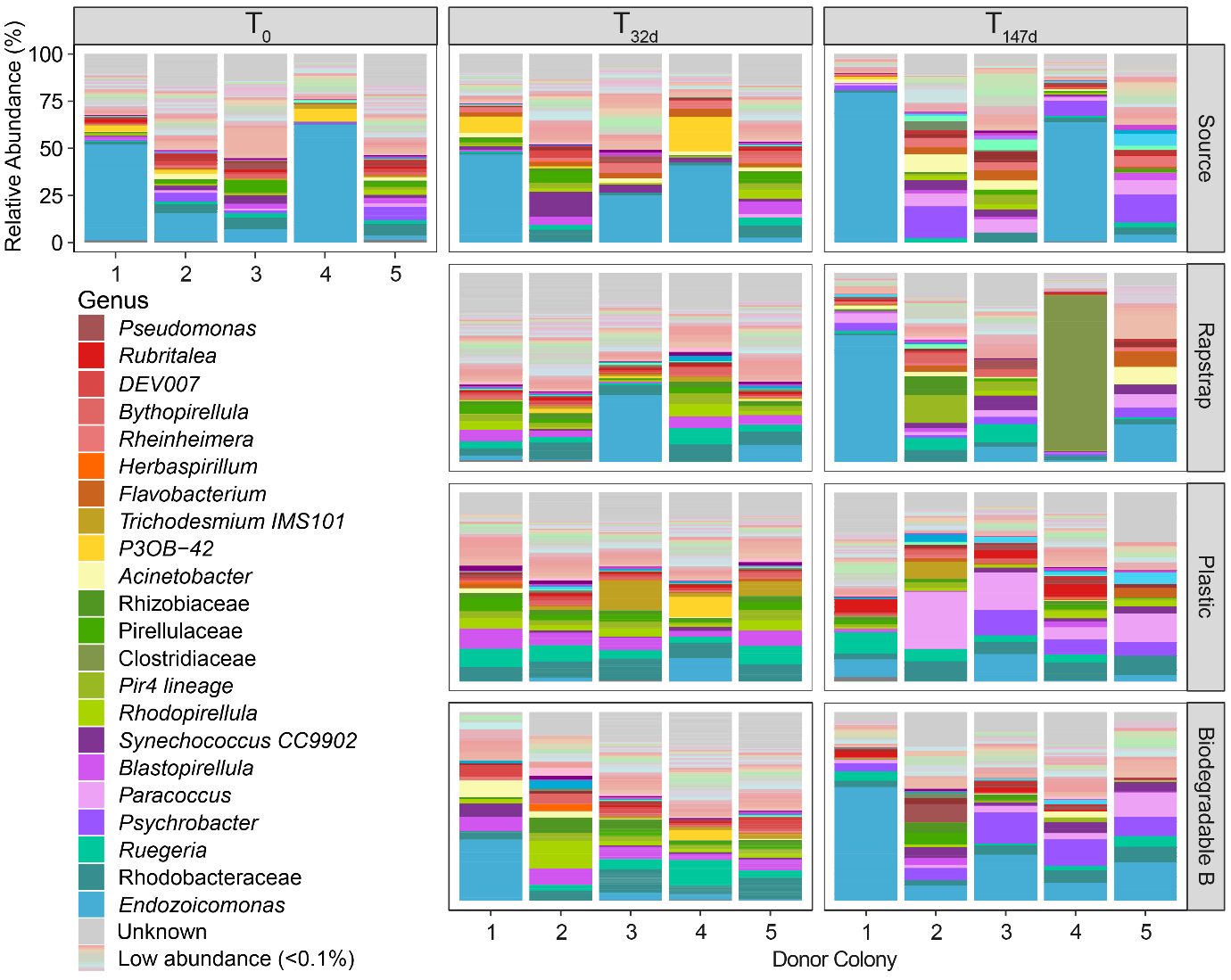


**Fig S6.** *Bacterial community composition (relative abundances) by genus* of donor colonies at the start of the experiment (T_0_) and fragments after 32 and 147 days (from February to August 2022) within a nursery secured with different materials; Rapstrap, plastic and biodegradable material B. Pastel colours represent genera with an average relative abundance of < 0.1 % in all samples, full legend provided as supplemental data (Supplementary Data S3b.) *non-italic text indicates family – where taxonomic resolution to genus level was unavailable.*

## Supplementary Tables

| **Experiment 1** | | |
| --- | --- | --- |
| **Material Details** | | |
| **Biodegradable A**  **(gocableties.co.uk)** | **Metal** | **Plastic** |
| SES-QUICK BIO biodegradable polyester (partially biobased of wheat or sugar cane).  Poly 1,4-butanediol Succinate – PBS.  300 mm x 7.6 mm | 316 Grade stainless steel.  200 mm x 4.6 mm | Nylon 66 – polyamide.  Black colour.  300 mm x 4.8 mm |
| 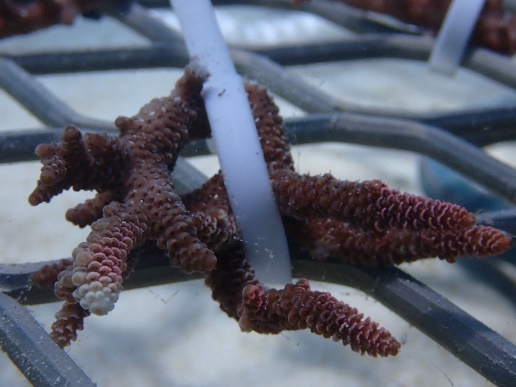 | 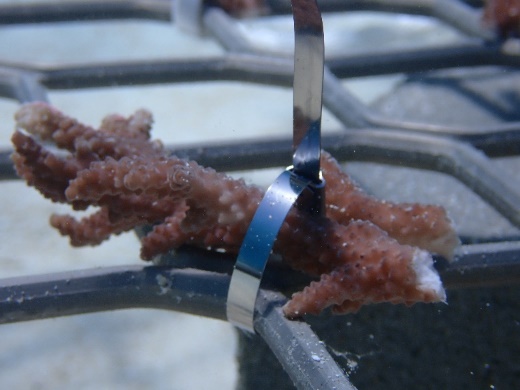 | 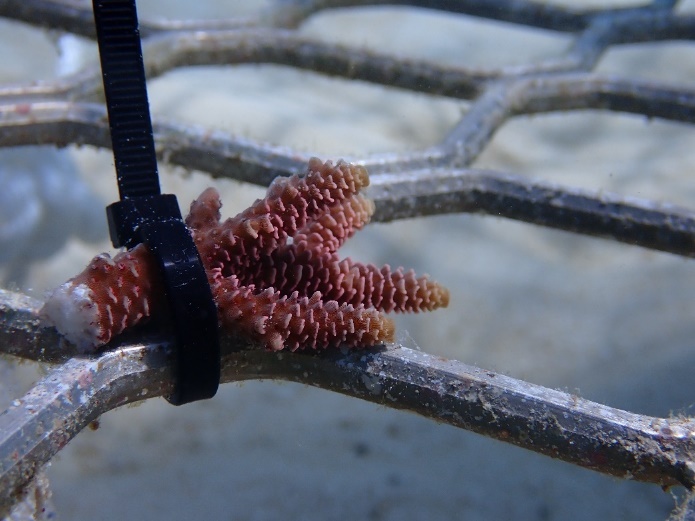 |
| **Experiment 2** | | |
| **Material Details** | | |
| **Biodegradable B**  **(rapstrap.com)** | **Rapstrap** (i-Tie: 052-MPU)  **(rapstrap.com)** | **Plastic** |
| Polycaprolactone  300 mm x 8 mm | Polyurethane Elastomer.  300 mm x 4.8 mm | Nylon 66 – polyamide. “Natural” colour.  300 mm x 4.8 mm |
| 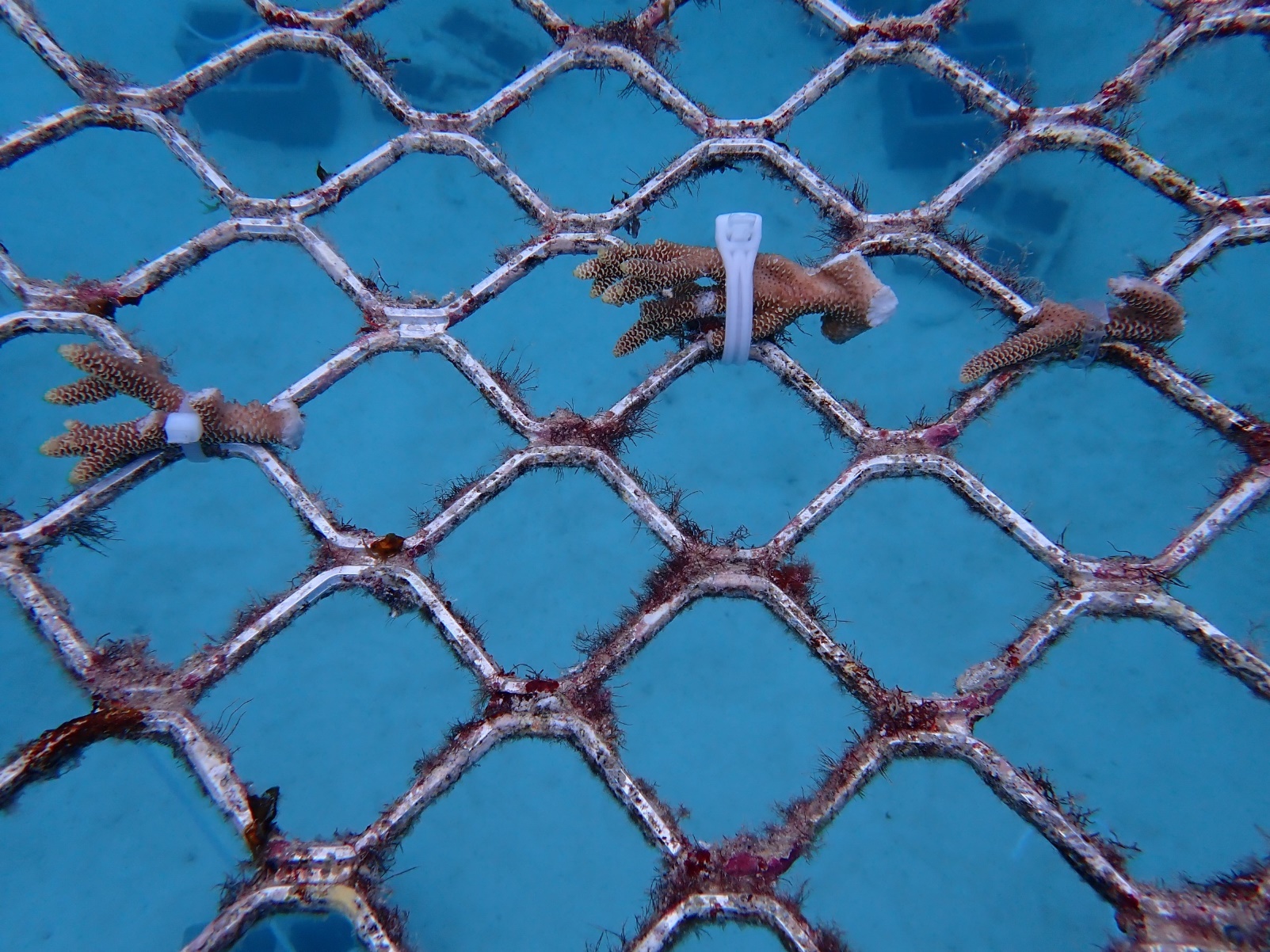 | 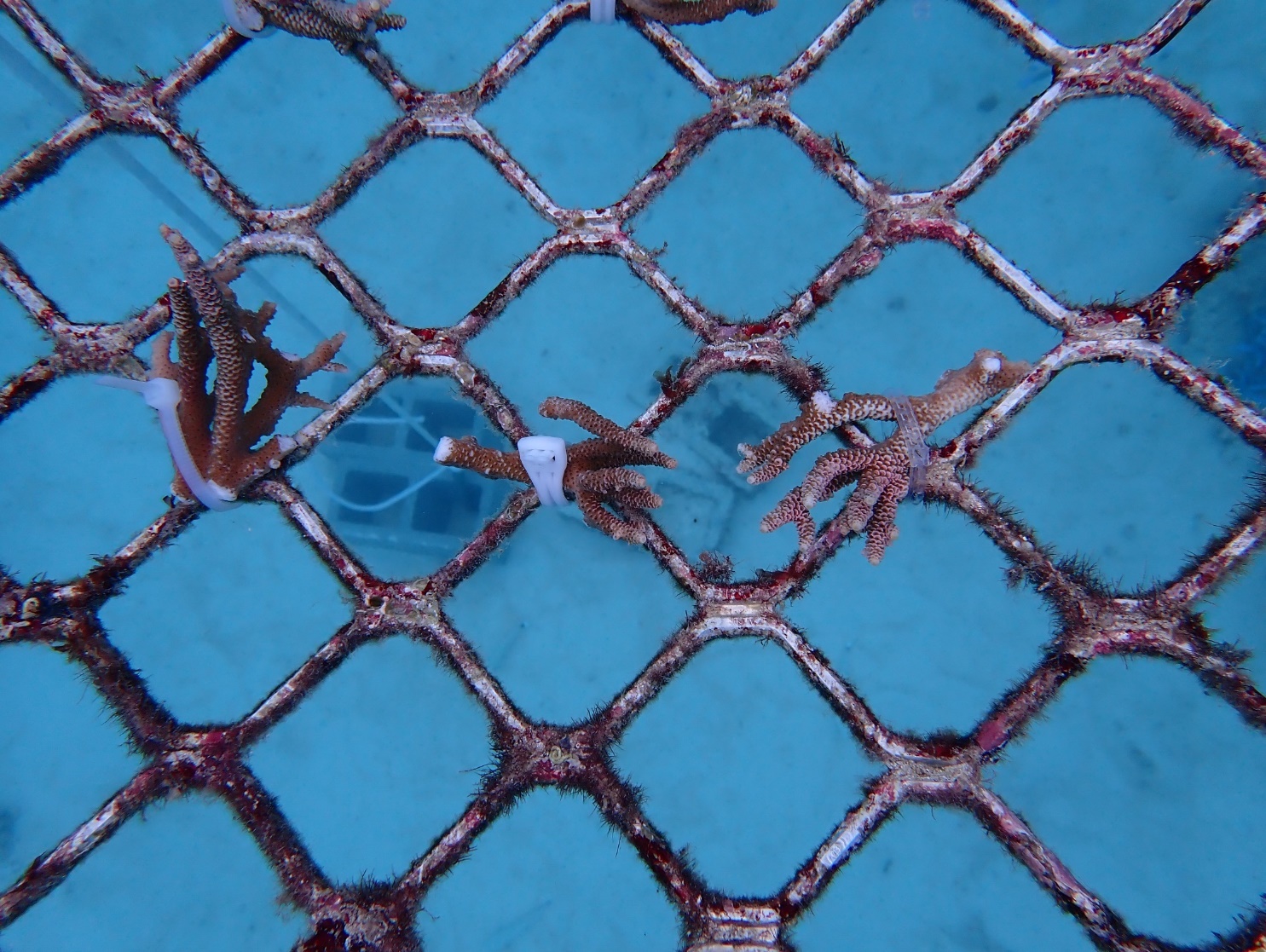 | 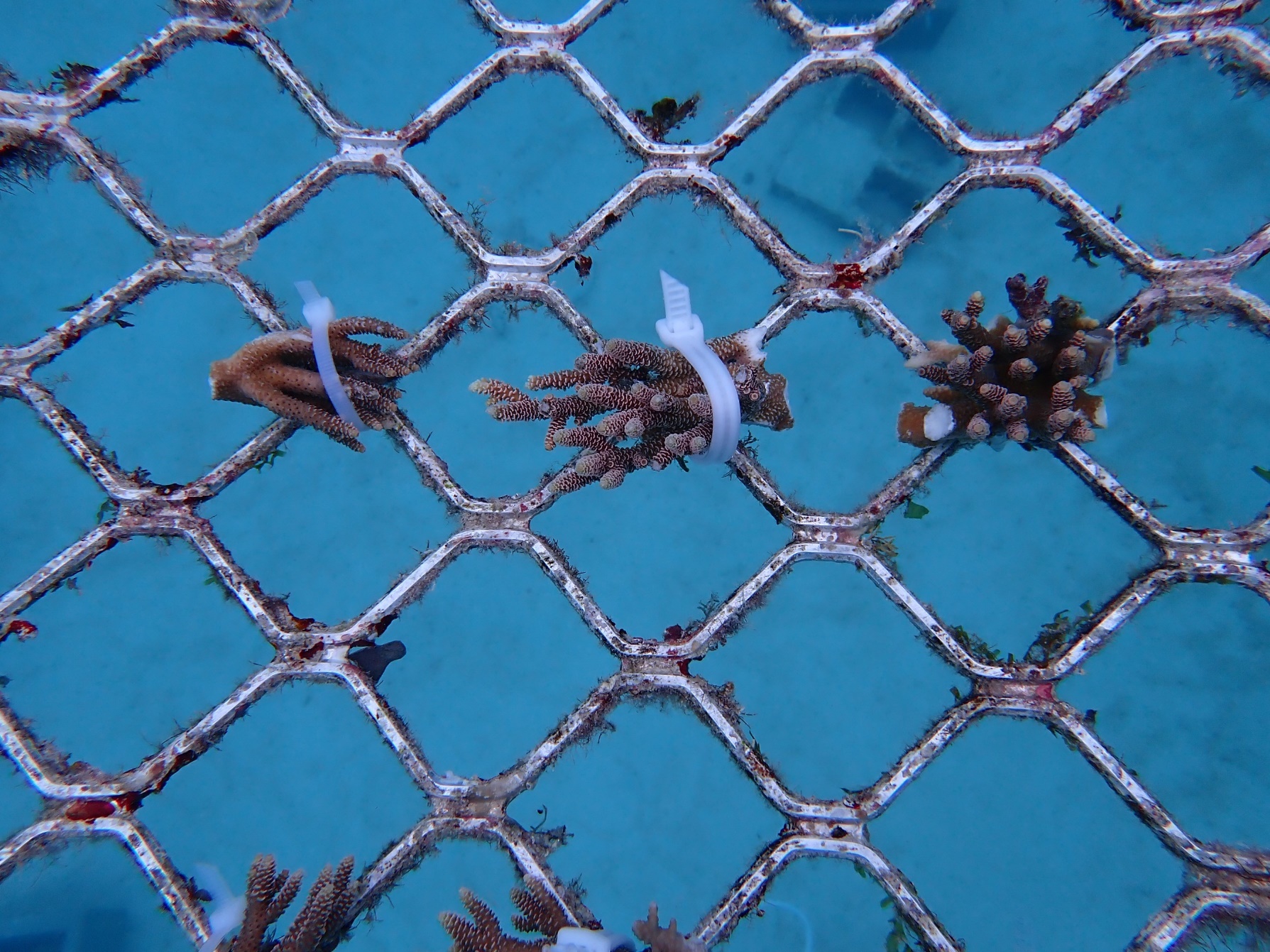 |

**Table S1.** *Details of the attachment materials used in each experiment with photographs of corals attached to nursery frames.*

| **Failure** | **Photograph, Experiment Number and Tie Details.** | |
| --- | --- | --- |
| Missing coral fragment and tie. | 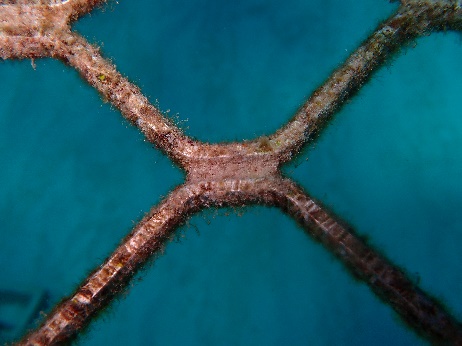  Experiment 1; Biodegradable (A). |  |
| Missing tie and coral fragment still present. | 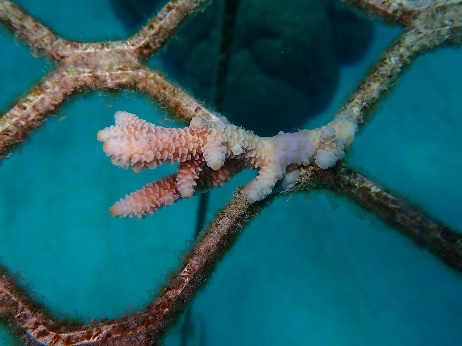  Experiment 1; Biodegradable (A). | 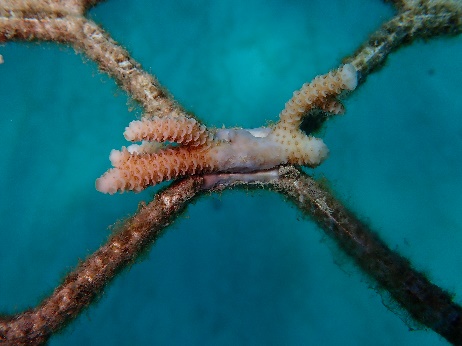  Experiment 1; Biodegradable (A). |
| Dead corals | 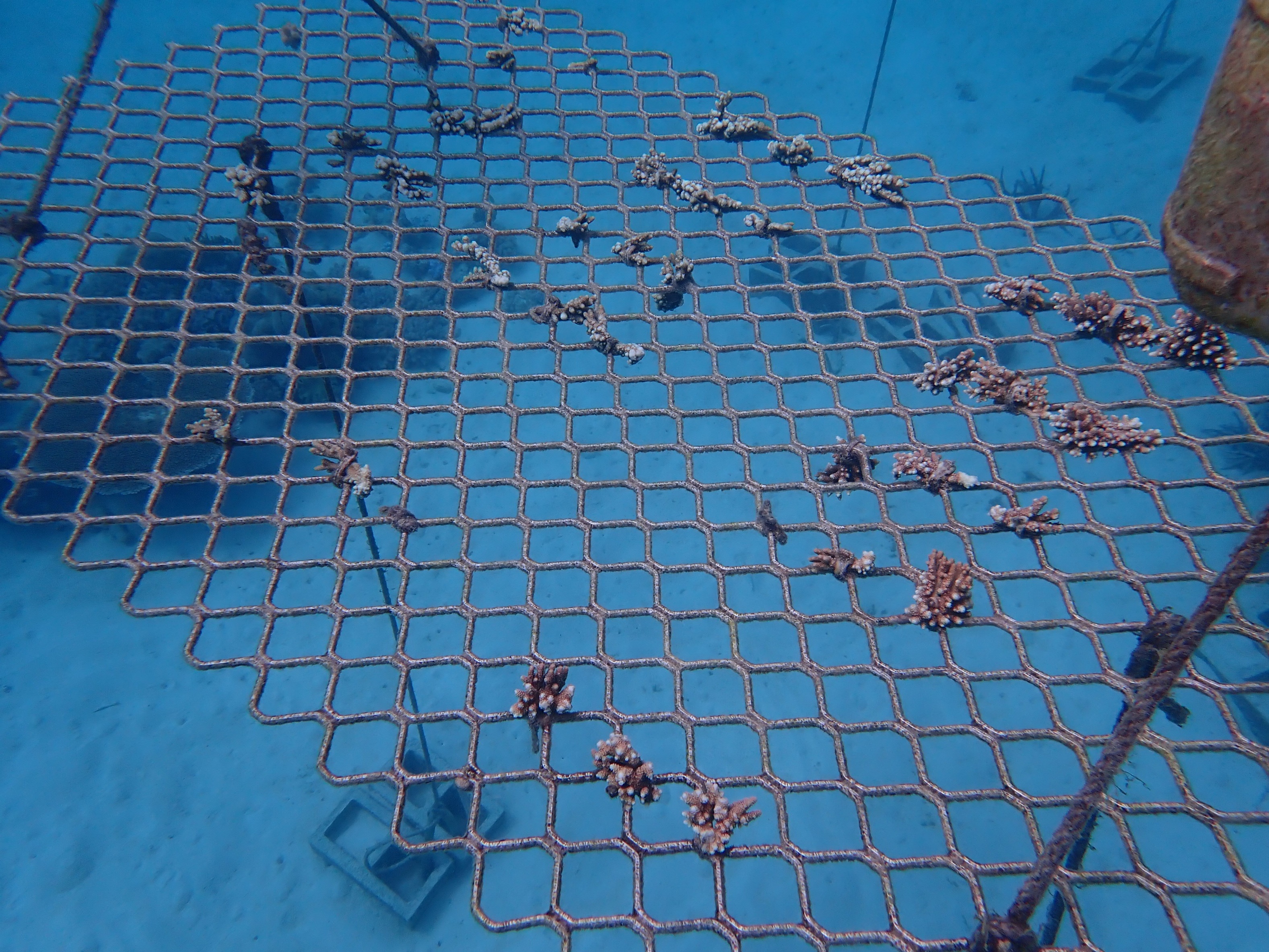  Experiment 2; Plastic tie. | 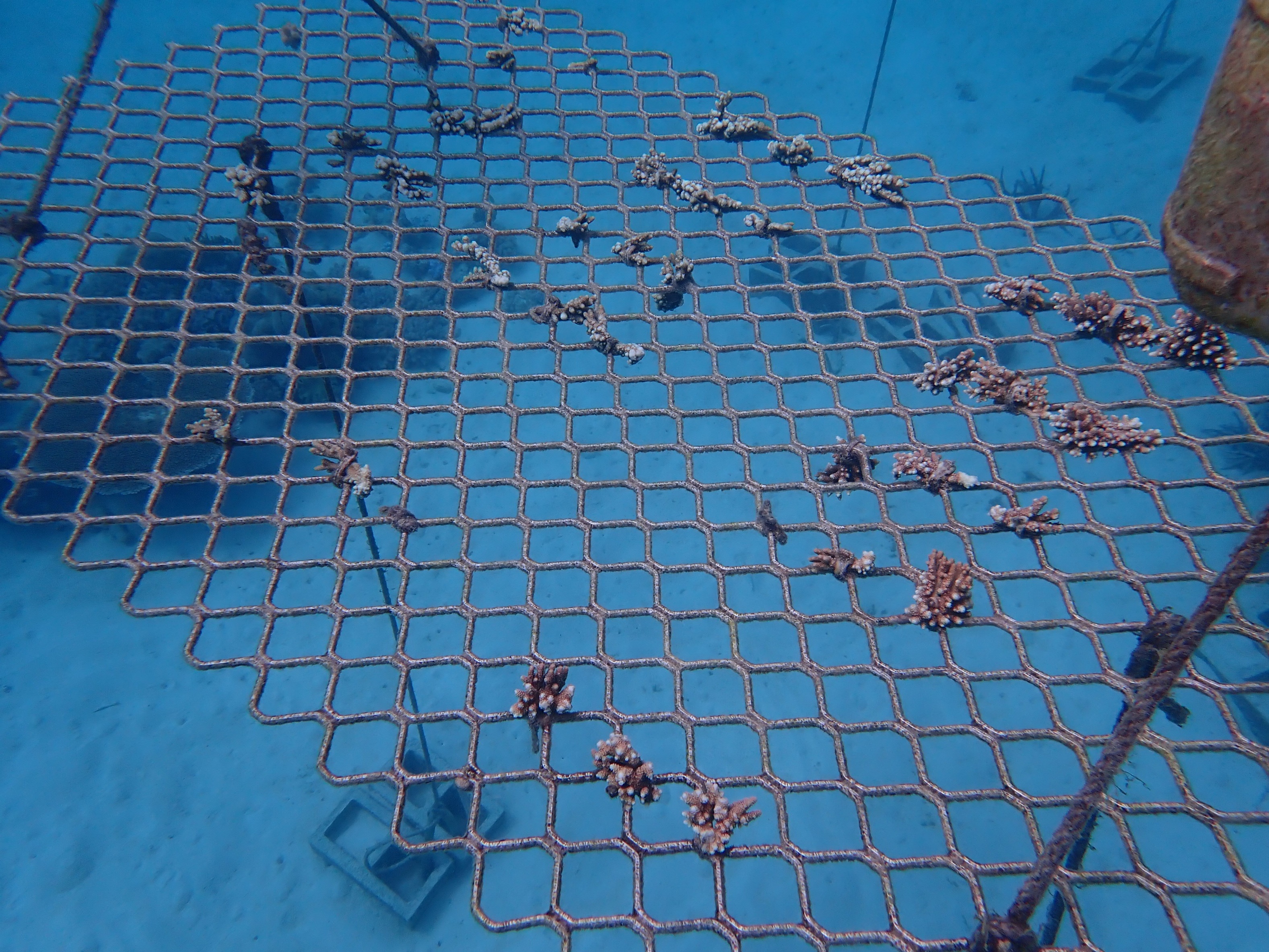  Experiment 2; Rapstrap tie. |

**Table S2.** *Examples of tie failure and coral mortality­.*

| Experiment 1 | | | | | | | | | |
| --- | --- | --- | --- | --- | --- | --- | --- | --- | --- |
| Timepoint | T_0_ | | | T_56d_ | | | T_189d_ | | |
|  | Metal | Bio A | Plastic | Metal | Bio A | Plastic | Metal | Bio A | Plastic |
| attached with tie | 24 | 24 | 24 | 24 | 21 | 24 | 22 | 11 | 22 |
| missing completely | 0 | 0 | 0 | 0 | 1 | 0 | 0 | 5 | 0 |
| coral missing tie present | 0 | 0 | 0 | 0 | 0 | 0 | 1 | 0 | 1 |
| coral present tie missing | 0 | 0 | 0 | 0 | 2 | 0 | 0 | 1 | 0 |
| dead coral | 0 | 0 | 0 | 0 | 0 | 0 | 0 | 0 | 0 |
| *expected coral fragments* | *24* | *24* | *24* | *24* | *24* | *24* | *23* | *18* | *23* |
|  |  |  |  |  |  |  | Metal | Bio A | Plastic |
|  |  |  |  |  |  | total failure % | 4.166667 | 29.16667 | 4.166667 |
| Experiment 2 | | | | | | | | | |
| Timepoint | T_0_ | | | T_32d_ | | | T_147d_ | | |
|  | Rapstrap | Bio B | Plastic | Rapstrap | Bio B | Plastic | Rapstrap | Bio B | Plastic |
| attached with tie | 28 | 29 | 29 | 26 | 29 | 29 | 23 | 28 | 27 |
| missing completely | 0 | 0 | 0 | 0 | 0 | 0 | 0 | 0 | 0 |
| coral missing tie present | 0 | 0 | 0 | 0 | 0 | 0 | 0 | 0 | 0 |
| coral present tie missing | 0 | 0 | 0 | 0 | 0 | 0 | 0 | 0 | 0 |
| dead coral | 0 | 0 | 0 | 2 | 0 | 0 | 2 | 0 | 1 |
| *expected coral fragments* | *28* | *29* | *29* | *28* | *29* | *29* | *25* | *28* | *28* |
|  |  |  |  |  |  |  | Rapstrap | Bio B | Plastic |
|  |  |  |  |  |  | total failure % | 0 | 0 | 0 |

**Table S3.** *Survivorship and failure counts for experiment 1 and 2 at sampling time points.*
